# *Mycobacterium tuberculosis* Bacteremia in a Cohort of HIV-Infected Patients Hospitalized with Severe Sepsis in Uganda–High Frequency, Low Clinical Sand Derivation of a Clinical Prediction Score

**DOI:** 10.1371/journal.pone.0070305

**Published:** 2013-08-05

**Authors:** Shevin T. Jacob, Patricia B. Pavlinac, Lydia Nakiyingi, Patrick Banura, Jared M. Baeten, Karen Morgan, Amalia Magaret, Yuka Manabe, Steven J. Reynolds, W. Conrad Liles, Anna Wald, Moses L. Joloba, Harriet Mayanja-Kizza, W. Michael Scheld

**Affiliations:** 1 University of Washington, Department of Medicine, Seattle, Washington, United States of America; 2 University of Washington, International Respiratory and Severe Illness Center (INTERSECT), Seattle, Washington, United States of America; 3 University of Washington, Department of Epidemiology, Seattle, Washington, United States of America; 4 Makerere University, College of Health Sciences, School of Medicine, Kampala, Uganda; 5 Makerere University, Infectious Diseases Institute, Kampala, Uganda; 6 Masaka Regional Referral Hospital, Masaka, Uganda; 7 University of Washington, Department of Global Health, Seattle, Washington, United States of America; 8 Joint Clinical Research Centre, JCRC TB Laboratory, Mengo, Uganda; 9 University of Washington, Department of Laboratory Medicine, Seattle, Washington, United States of America; 10 Fred Hutchinson Cancer Research Center, Vaccine and Infectious Disease Division, Seattle, Washington, United States of America; 11 Johns Hopkins University, School of Medicine, Baltimore, Maryland, United States of America; 12 Rakai Health Sciences Program, Kalisizo, Uganda; 13 National Institutes of Health, Department of Intramural Research, Bethesda, Maryland, United States of America; 14 Makerere University, Department of Medical Microbiology, Kampala, Uganda; 15 University of Virginia, Department of Medicine, Charlottesville, Virginia, United States of America; National Institute of Infectious Diseases, Japan

## Abstract

**Background:**

When manifested as *Mycobacterium tuberculosis* (MTB) bacteremia, disseminated MTB infection clinically mimics other serious blood stream infections often hindering early diagnosis and initiation of potentially life-saving anti-tuberculosis therapy. In a cohort of hospitalized HIV-infected Ugandan patients with severe sepsis, we report the frequency, management and outcomes of patients with MTB bacteremia and propose a risk score based on clinical predictors of MTB bacteremia.

**Methods:**

We prospectively enrolled adult patients with severe sepsis at two Ugandan hospitals and obtained blood cultures for MTB identification. Multivariable logistic regression modeling was used to determine predictors of MTB bacteremia and to inform the stratification of patients into MTB bacteremia risk categories based on relevant patient characteristics.

**Results:**

Among 368 HIV-infected patients with a syndrome of severe sepsis, eighty-six (23%) had MTB bacteremia. Patients with MTB bacteremia had a significantly lower median CD4 count (17 vs 64 lymphocytes/mm^3^, p<0.001) and a higher 30-day mortality (53% vs 32%, p = 0.001) than patients without MTB bacteremia. A minority of patients with MTB bacteremia underwent standard MTB diagnostic testing (24%) or received empiric anti-tuberculosis therapy (15%). Independent factors associated with MTB bacteremia included male sex, increased heart rate, low CD4 count, absence of highly active anti-retroviral therapy, chief complaint of fever, low serum sodium and low hemoglobin. A risk score derived from a model containing these independent predictors had good predictive accuracy [area under the curve = 0.85, 95% CI 0.80–0.89].

**Conclusions:**

Nearly 1 in 4 adult HIV-infected patients hospitalized with severe sepsis in 2 Ugandan hospitals had MTB bacteremia. Among patients in whom MTB was suspected, standard tests for diagnosing pulmonary MTB were inaccurate for correctly classifying patients with or without bloodstream MTB infection. A MTB bacteremia risk score can improve early diagnosis of MTB bacteremia particularly in settings with increased HIV and MTB co-infection.

## Introduction

In 2011, approximately one quarter of new *Mycobacterium tuberculosis* (MTB) cases worldwide occurred in sub-Saharan Africa where the tuberculosis epidemic is fueled by a high prevalence of HIV infection [1], [2]. In this region, MTB is the leading cause of death among HIV-infected persons and post-mortem studies have shown that a large proportion of those who die of MTB infection have undiagnosed disseminated disease [3], [4].

Several studies from sub-Saharan Africa have reported a high frequency of MTB bacteremia, a manifestation of disseminated tuberculosis, primarily among patients co-infected with HIV [5]–[11]. Similar to severe bloodstream infections caused by pathogens other than MTB, MTB bacteremia can clinically manifest as septic shock [12]–[14]. Along with the several week delay for results from standard mycobacterial culture methods, this non-specific presentation for MTB bacteremia contributes to difficulty in early diagnosis and ineffective empiric antimicrobial therapy during the early stages of illness. Moreover, since global efforts to measure the global burden and mortality of MTB focus on pulmonary rather than disseminated MTB disease, these case definitions often lack the sensitivity to accurately capture cases of MTB bacteremia leading to poor recognition of this manifestation of MTB infection by clinical providers [15].

An improved understanding of the clinical diagnosis and management of MTB bacteremia is needed for settings where the prevalence of HIV and MTB is high. In a prospective study of HIV-infected patients hospitalized with severe sepsis in Uganda, we assessed the frequency, clinical presentation and survival of patients with MTB bacteremia. In addition, we estimated clinical suspicion for diagnosing MTB bacteremia by determining how frequently clinicians utilized diagnostics and treatment for pulmonary MTB and we developed a risk score to assist clinicians in early identification of MTB bacteremia.

## Methods

### Ethics Statement

Ethical approval was obtained from the research and/or ethics committees of the University of Virginia, Makerere University, Mulago Hospital, Infectious Disease Institute, and the Uganda National Council of Science and Technology. Written informed consent was obtained from each patient or a surrogate if the patient was too obtunded to provide consent.

### Study Participants

Between May, 2008 and May, 2009, 426 adult (age ≥18 years) patients admitted with severe sepsis to the medical wards of Mulago National Referral Hospital in Kampala, Uganda, and Masaka Regional Referral Hospital in Masaka, Uganda, were enrolled in an intervention study of fluid resuscitation. Patients were included if they fulfilled the following modified criteria for severe sepsis: 1) suspected infection as determined by the admitting medical officer; 2) two of the following: a) axillary temperature >37.5 degrees Celsius or <35.5 degrees Celsius; b) heart rate >90 beats/min; c) respiratory rate >20 breaths/min; 3) systolic blood pressure ≤100 mmHg; and 4) whole blood lactate concentration >2.5 mmol/L or Karnofsky Performance Scale (KPS) score ≤40. Of this larger cohort, 368 patients were infected with HIV-1 and are the focus of this manuscript. Further details of inclusion criteria, site descriptions and assessment of the primary endpoint (30-day mortality) have been described elsewhere [14], [16].

### Clinical and Laboratory Evaluation

Clinical history (including chief complaint, duration of illness and history of treatment for active pulmonary MTB disease) and patient management data [including the results of diagnostic tests like chest radiograph and sputum acid fast bacilli (AFB) smear] were systematically noted in the evaluation. Blood samples were collected at the time of enrollment for complete blood counts, electrolytes, CD4+ T-cell (CD4) counts, HIV serology, malaria blood smears and blood cultures (both aerobic and mycobacterial). Blood cultures were performed using strict adherence to aseptic technique and prior to the administration of antimicrobial agents. Mycobacterial blood cultures from both study sites were performed in the mycobacteriology laboratory at the Joint Clinical Research Center in Kampala, Uganda.

### Mycobacterial Blood Cultures

For the optimal recovery of mycobacteria, 3–5 mL of blood were aseptically inoculated into Bactec Myco/F Lytic medium vials and incubated in the fluorescent series Bactec 9120 instrument [Becton Dickinson, Sparks, USA] at 35°C. Ziehl-Neelsen (ZN) smears performed on blood from positive vials were examined for AFB and morphological features (e.g., cording) of MTB. Aliquots of AFB positive vials were subcultured on blood agar and incubated for 48 hours at 35°C to assess for growth of possible non-mycobacterial organisms. AFB positive blood cultures were subcultured to 7H10 nonselective plates and 7H10 selective plates (with amphotericin B, carbenicillin, trimethoprim, and nalidixic acid added) for mycobacterial growth. MTB complex was identified using a polymerase chain reaction (PCR) assay to amplify the IS6110 target insertion sequence of MTB or by the Capilia TB assay [Nippon Becton Dickinson Co., Ltd, Tokyo, Japan] for the detection of the MTB-related MPB64 protein [17]; AFB positive but IS6110 or Capilia negative cultures were identified as non-tuberculous mycobacteria. To account for potential PCR inhibitors often found in blood culture media [18], IS6110 and Capilia assays were rerun on 7H10 subcultures of samples with a preliminary negative MTB PCR test. MTB bacteremia frequency was defined as the proportion of enrolled patients with mycobacterial blood cultures positive for MTB as confirmed by either PCR or antigen test. Drug susceptibility testing for first-line anti-tuberculosis therapy was conducted on Mycobacterial Growth Indicator Tube subcultures inoculated from 0.2 mL of pure MTB blood cultures or from colonies of MTB grown on 7H10 plates.

### Statistical Analysis

Statistical analyses were conducted using SPSS 17.0 (SPSS Inc., Somers, USA) and Stata 10.1 (Stata Corp., TX, USA). Predictors of MTB bacteremia were determined using univariate logistic regression with robust standard errors. Potential predictors were evaluated from patient demographics, admission vital signs, characteristics of HIV infection, clinical descriptors and laboratory blood tests.

Cox proportional hazards regression was used to evaluate the time from hospital admission until death. Patients lost to follow-up before 30 days contributed follow-up time until their last known assessment of vital status and were censored thereafter. The crude associations between death and MTB bacteremia were estimated by hazard ratios (HR) and 95% confidence intervals (CI). Potential confounders were included stepwise in a multivariable model and remained in the model if the adjusted association differed from the unadjusted hazard ratio by more than 10%. Kaplan–Meier estimates of mortality were compared between those with and without MTB bacteremia using the log-rank test.

To estimate suspicion for MTB bacteremia by clinicians caring for study patients, we determined the frequency at which standard MTB diagnostic tests (i.e., chest radiograph and AFB sputum smear) were ordered and empiric anti-tuberculosis therapy was administered. Chest radiographs were defined as abnormal if there were findings of infiltrate, effusion, miliary pattern or cavitary lesion. Results of AFB smears were provided by hospital laboratory technicians. Performance characteristics of standard MTB diagnostic tests for diagnosing MTB bacteremia were calculated. For patients with missing standard MTB diagnostic test results, sensitivity analyses were performed to evaluate the performance characteristics of these tests for MTB bacteremia in scenarios where either all of these patients were assumed to have a positive AFB smear and chest radiograph or all of them were assumed to have a negative AFB smear and chest radiograph. Finally, backwards stepwise regression was used to assess factors associated with receiving anti-tuberculosis therapy among patients with MTB bacteremia. Predictors with univariate p-values <0.20 were included in the full model and removed stepwise using a p-value ≤0.05 until a final parsimonious model was achieved.

Using the same p-value criteria to achieve a final model as described above, backwards stepwise regression was also utilized for development of a multivariable prediction model of MTB bacteremia among patients with complete data. Model-fit was assessed by the area under the curve (AUC) and associated bootstrapped 95% CI. The final model was validated by 10-fold cross-validation and predictive accuracy reported as the mean, minimum and maximum AUC generated from the 10 iterations [19]. To determine the incremental benefit of the final model over a model comprised of only HIV-associated predictors and a model comprised of non-laboratory dependent characteristics, the AUCs of the three models were compared using non-parametric methods based on a generalized *U-*statistic [20].

To adapt the multivariable prediction model for MTB bacteremia into a tool for clinical use we developed a risk score to estimate probability of MTB bacteremia based on previously described methodology [21]. Variables from the final multivariable prediction model were stratified according to clinically relevant cut-offs [22] and strata with the lowest risk for MTB bacteremia were designated as the referent (Methods S1, Table S1). Coefficients from the final multivariable prediction model were used to calculate score values for each variable stratum. Risk scores were categorized according to estimated probability of having MTB bacteremia: low (<30%), moderate (30–70%) and high (>70%).

## Results

### Frequency and Characterization of MTB Bacteremia

**Figure 1:**
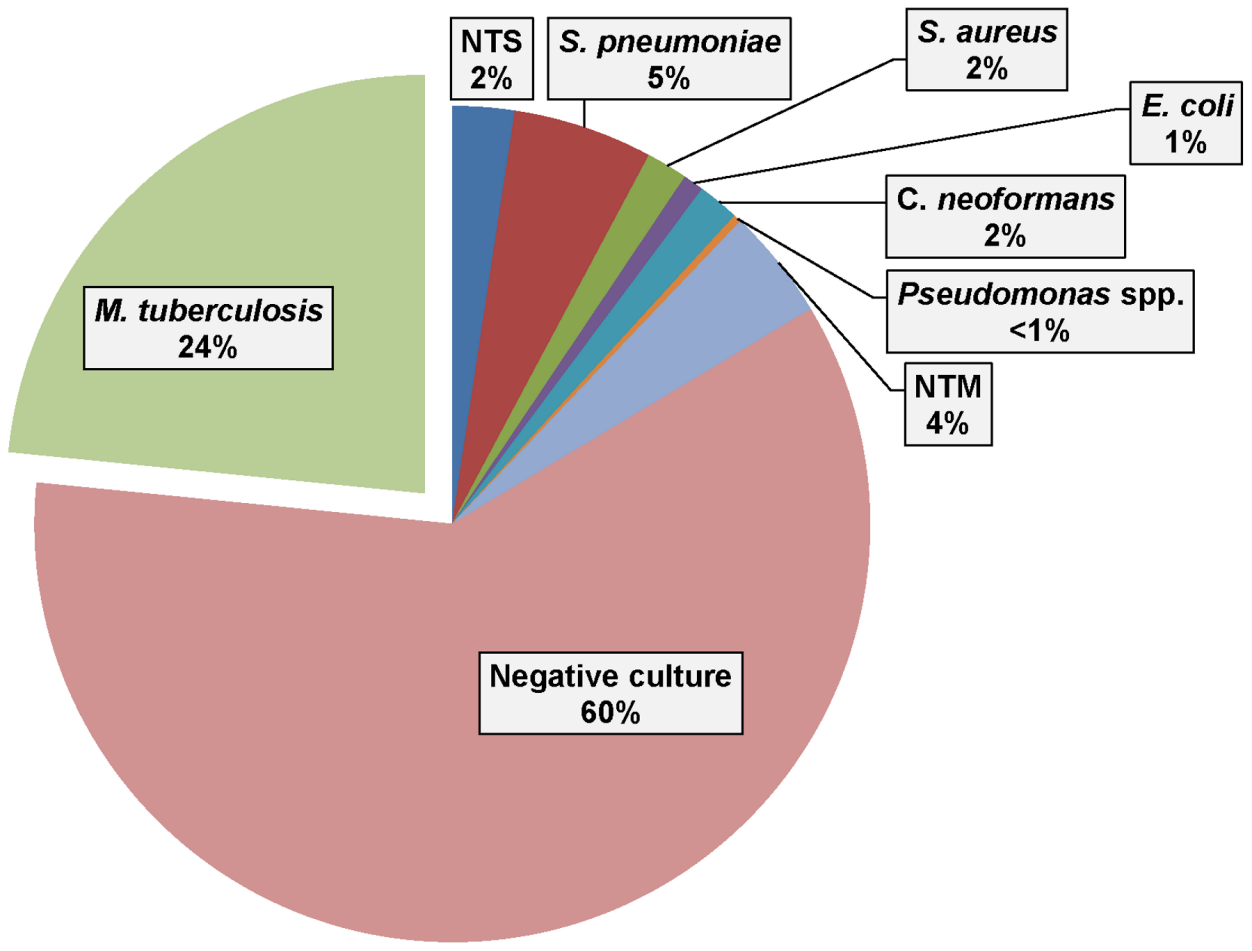
Prevalence of MTB in blood culture compared to other causes of bacteremia among HIV-infected patients (n = 368). [NTS = non-typhoidal Salmonella; NTM = non-tuberculous mycobacteria].

Among 368 HIV-infected patients, MTB bacteremia was detected in 86 (23.4%) and was more common than bacteremia from any other organism; non-tuberculous mycobacteria was isolated in 4% of blood cultures (Figure 1). Eight patients with MTB bacteremia also had bacterial growth on blood agar plates but all such isolates were deemed to be contaminants. Twelve (14%) of 86 patients with MTB drug susceptibility testing had resistance (5 to streptomycin, 8 to isoniazid, and 2 to rifampin); none fulfilled criteria for multi-drug or extensive-drug resistance.

### Comparison of Baseline Characteristics between Patients with and without MTB Bacteremia

Overall, baseline characteristics of HIV-infected patients with MTB bacteremia were similar to patients without MTB bacteremia (Table 1). Compared to patients without MTB bacteremia, however, patients with MTB bacteremia had a significantly lower median CD4 count (17 vs 64 lymphocytes/mm^3^, p<0.001) and fewer were taking highly active anti-retroviral therapy (HAART) at the time of hospitalization (92% vs 72%, p<0.001) (Table 2). Additionally, a significantly lower proportion of patients with MTB bacteremia had received prior therapy for active pulmonary MTB disease (7% vs 22%, p = 0.005). Significantly more patients with MTB bacteremia presented with a persistent fever (46% vs 34%, p = 0.02) and a longer duration of illness prior to hospitalization (median 30 vs 14 days, p = 0.05). Also, significantly more patients with MTB bacteremia had elevated lactate concentrations (4.1 vs 3.7 mmol/L, p = 0.02) and lower median hemoglobin (7.5 vs 9.3 g/dL, p<0.001), serum sodium (128 vs 131 mEq/L, p<0.001) and serum albumin (2.3 vs 2.6 g/L, p<0.001) concentrations.

**Table 1:** Baseline characteristics in HIV-infected patients with MTB bacteremia compared to patients without MTB bacteremia.

Characteristic	Study population(n = 368)^a^	MTB bacteremia(n = 86)^a^	No MTB bacteremia (n = 282)^a^
***Demographics:***			
Age in years [median (IQR)]	34 (27–40)	35 (27–39)	34 (27–40)
Male (vs. Female) [n (%)]	174 (47.3)	46 (53.5)	128 (45.4)
***Admission vital signs:***			
Temperature, °Celsius [median (IQR)]	37.9 (36.9–38.9)	37.8 (36.9–39)	37.9 (36.9–38.9)
Heart rate, beats/min [median (IQR)]	128 (113.2–144)	136 (120–148)	127 (112–141)
Respiratory rate, breaths/min [median (IQR)]	36 (28–42)	37 (30–44)	36 (28–42)
Systolic blood pressure, mmHg [median (IQR)]	85 (78–90)	80 (70–90)	86 (78–90)
% oxygen saturation [median (IQR)]	96 (94–98)	96 (94–98)	96 (94–98)
***HIV descriptors:***			
CD4 count, lymphocytes/mm^3^ [median (IQR)]	46 (13–128.8)	17 (5–47)	64 (15–145.5)
Unaware of HIV status [n (%)]	120 (33.1)	27 (31.8)	93 (33.5)
Not taking TMP-SMX prophylaxis [n (%)]	130 (35.3)	34 (39.5)	96 (34.0)
Not taking HAART [n (%)]	282 (76.6)	79 (91.9)	203 (72.0)
***Chief complaint:***			
Fever [n (%)]	133 (36.1)	40 (46.5)	93 (33.0)
Dyspnea [n (%)]	27 (7.3)	2 (2.3)	25 (8.9)
Cough [n (%)]	78 (21.2)	17 (19.8)	61 (21.6)
Chest pain [n (%)]	11 (3.0)	2 (2.3)	9 (3.2)
***Other clinical variables:***			
Duration of illness prior to hospitalization, days [median (IQR)]	17.5 (7–30)	30 (14–60)	14 (7–30)
Treatment for MTB prior to hospitalization [n (%)]	65 (18.2)	6 (7.2)	59 (21.5)
Admit Karnofsky Performance Score [median (IQR)]	40 (30–50)	40 (30–50)	40 (30–50)
***Laboratory variables:***			
Lactate, mmol/L [median (IQR)]	3.9 (3.0–4.8)	4.1 (3.5–5.5)	3.7 (3–4.7)
White blood cell count, cells/mL [median (IQR)]	4500 (2800–7900)	4600 (3100–7900)	4500 (2700–7975)
Platelets, cells/mL [median (IQR)]	193,000 (109,000–299,000)	172,500 (99,250–281,250)	198,000 (112,500–302,000)
Hemoglobin, g/dL [median (IQR)]	8.9 (7.4–10.8)	7.4 (6.5–9.1)	9.3 (7.9–11.2)
Sodium, mEq/L [median (IQR)]	130 (127–135)	128 (123–131)	131 (128–135)
Potassium, mEq/L [median (IQR)]	3.8 (3.4–4.3)	3.9 (3.3–4.3)	3.8 (3.4–4.3)
Bicarbonate, mEq/L [median (IQR)]	19 (17–22)	18 (15–20)	20 (17–22)
Blood urea nitrogen, mg/dL [median (IQR)]	13.8 (8–25)	17 (10–32)	12 (7–24)
Creatinine, mg/dL [median (IQR)]	1.0 (0.8–1.7)	1.1 (0.8–2.0)	1.0 (0.7–1.7)
Glucose, mg/dL [median (IQR)]	105 (89.5–121.5)	106 (94–126)	104 (88–119)
Albumin, g/L [median (IQR)]	2.5 (2.1–2.9)	2.3 (2.0–2.5)	2.6 (2.1–3.0)

a Due to the occurrence of missing data found in <5% of the variables, numbers may not add up to total n.

[abbreviations: MTB = *Mycobacterium tuberculosis*; IQR = inter-quartile range; CD4 = CD4+ T-cell count; TMP-SMX = trimethoprim-sulfamethoxazole; HAART = highly active anti-retroviral therapy].

**Table 2:** Univariate and multivariate analysis of clinical predictors for MTB bacteremia in HIV-infected patients with severe sepsis.

Characteristic	Univariate Analysis(OR, 95% CI)	p-value	Multivariate Analysis(OR, 95% CI)	p-value
***Demographics:***				
Age (years)	1.00 (0.98–1.03)	0.9		
Male (vs. Female)	1.38 (0.85–2.25)	0.2	2.88 (1.40,5.91)	0.004
***Admission vital signs:***				
Temperature	0.97 (0.83–1.14)	0.7		
Heart rate per 10 beats per minute	1.20 (1.06–1.36)	0.003	1.18 (1.00,1.40)	0.046
Respiratory rate	1.01 (0.99–1.03)	0.4		
Systolic blood pressure	0.99 (0.97–1.01)	0.2	–	
Oxygen saturation	1.01 (0.97–1.06)	0.6		
***HIV descriptors:***				
CD4 count per 100 lymphocytes/mm^3^	0.34 (0.21–0.54)	<0.001	0.32 (0.17,0.60)	<0.001
Unaware of HIV status	1.13 (0.62–2.06)	0.7		
Not taking TMP-SMX prophylaxis at time of admission	1.27 (0.77–2.08)	0.4		
Not taking HAART at time of admission	4.39 (1.94–9.93)	<0.001	8.65 (2.66,28.19)	<0.001
***Chief complaint:***				
Fever	1.77 (1.08–2.89)	0.02	1.99 (1.01, 3.92)	0.045
Dyspnea	0.24 (0.057–1.06)	0.06	–	
Cough	0.89 (0.49–1.63)	0.7		
Chest pain	0.72 (0.15–3.41)	0.7		
***Other clinical variables:***				
Duration of illness prior to hospitalization (days)	1.01 (1.00–1.01)	0.05	–	
Treatment for MTB prior to hospitalization	0.28 (0.12–0.69)	0.005	–	
Admit Karnofsky Performance Score	1.00 (0.98–1.02)	1.0		
***Laboratory variables:***				
Lactate	1.16 (1.02–1.30)	0.02	–	
White blood cell count	1.00 (1.00–1.00)	0.5		
Platelets	1.30 (0.72–2.32)	0.4		
Hemoglobin	0.76 (0.68–0.85)	<0.001	0.76 (0.65,0.88)	<0.001
Sodium	0.91 (0.87–0.95)	<0.001	0.91 (0.86,0.97)	0.004
Potassium	0.91 (0.64–1.30)	0.6		
Bicarbonate	0.90 (0.85–0.96)	0.001	–	
Blood urea nitrogen	1.01 (1.00–1.02)	0.1	–	
Creatinine	0.99 (0.84–1.16)	0.9		
Glucose	1.007 (1.00–1.01)	0.06	–	
Albumin	0.90 (0.86–0.95)	<0.001	–	

[abbreviations: MTB = *Mycobacterium tuberculosis*; OR = odds ratio; CI = confidence interval; CD4 = CD4+ T-cell count; TMP-SMX = trimethoprim-sulfamethoxazole; HAART = highly active anti-retroviral therapy].

### Survival in Patients with MTB Bacteremia

**Figure 2:**
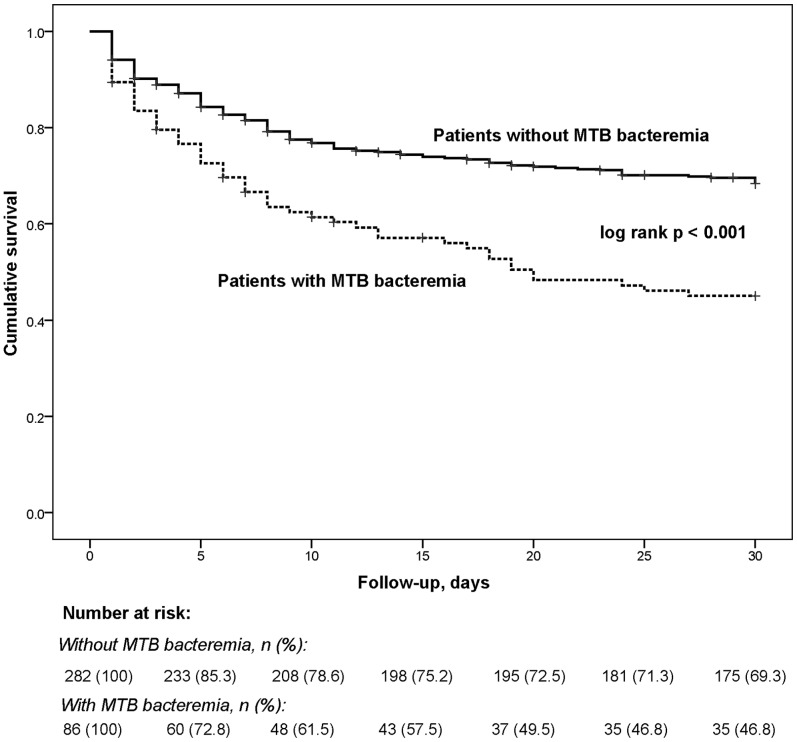
Kaplan-Meier survival curves comparing 30-day mortality between patients with MTB bacteremia and patients without MTB bacteremia.

Patients with MTB bacteremia had a significantly higher 30-day mortality compared to patients without MTB bacteremia [53.1% (43/81) vs. 31.9% (83/260), p = 0.001; unadjusted HR 2.0, 95% CI 1.4–2.9] (Figure 2); 7.3% (27/368) were lost to follow-up. Approximately 50% of patients with MTB bacteremia died within 18 days of admission, just prior to the median time when the results of mycobacterial cultures became available [19 days, inter-quartile range (IQR) 15–24]. The association between MTB bacteremia and 30-day mortality was similar after adjustment for confounders (CD4 count, absence of HAART, anti-tuberculosis therapy prior to hospitalization and serum albumin concentration) (adjusted HR 1.8, 95% CI 1.2–2.8, p = 0.003). Compared to patients with MTB bacteremia who did not receive empiric anti-tuberculosis therapy at the time of admission, a non-significant trend towards decreased 30-day mortality was noted among patients with MTB bacteremia treated with empiric anti-tuberculosis therapy [30.8% (4/13) vs. 53.4% (39/73), p = 0.1].

### Clinical Suspicion for MTB Bacteremia

Of all 368 patients, 25% had an AFB sputum smear and 53% had a chest radiograph ordered as part of the diagnostic work-up by the admitting clinician. Among 86 patients with confirmed MTB bacteremia, 26 (30.2%) received an AFB sputum smear and just over half (57%) underwent a chest radiograph; only 21 (24%) of 86 patients had both an AFB smear and chest radiograph ordered. While both AFB smear (77%) and chest radiograph (98%) were sensitive for diagnosing MTB bacteremia, specificity and positive predictive value for MTB bacteremia were poor (<65%) for both tests (Table 3). If all remaining patients with missing diagnostic test data were to have AFB sputum smear and chest radiographs performed and the results of these tests were assumed to be positive, the estimated sensitivity of smear and radiograph for MTB bacteremia would increase to 94.2% and 98.8%, respectively, while the corresponding estimated specificity would decrease (12.8% and 3.2%, respectively). On the other hand, if the same patients had the MTB diagnostic tests performed and all the results were assumed to be negative, sensitivity would decrease considerably for both smear (19.8%) and radiograph (53.5%) but estimated specificity would increase for both tests with AFB smear having a high specificity for MTB bacteremia (92.6%) while the specificity of the radiograph would remain low (53.2%).

**Table 3:** Performance characteristics for diagnosing MTB bacteremia using AFB sputum smear and chest radiograph.

Diagnostic test	Sensitivity of test forMTB bacteremia	Specificity of test for MTB bacteremia	Positive predictive value of test for MTB bacteremia	Negative predictive value of test for MTB bacteremia
AFB sputum smear (n = 79)*	17/22 (77.3%)	36/57 (63.2%)	17/38 (44.7%)	36/41 (87.8%)
Chest radiograph (n = 188)**	46/47 (97.9%)	9/141 (6.4%)	46/178 (25.8%)	9/10 (90.0%)

* Results from 13 collected AFB sputum smears were not available for this analysis.

** Results from 6 ordered chest radiographs were not available for this analysis.

[abbreviations: MTB = *Mycobacterium tuberculosis*; AFB = acid fast bacilli].

A minority (15%) of patients with MTB bacteremia received empiric anti-tuberculosis therapy within the first 24 hours of admission to treat suspected MTB infection. Multivariable analysis revealed that admitting clinicians were more likely to provide anti-tuberculosis therapy in patients with cough as a chief complaint (relative risk 4.9, 95% CI 1.2–19.4, p = 0.02); both hyperlactatemia and hyperkalemia were also associated with administration of anti-tuberculosis therapy but these covariates did not remain statistically significant in the final multivariable model (data not shown).

### Clinical Predictors and Risk Score for Diagnosing MTB Bacteremia

Two-hundred eighty-four patients (78%) had complete data for all considered predictors; no significant difference in proportion of missing variables existed between patients with and without MTB bacteremia (21% vs. 23%, p = 0.63). Independent factors associated with MTB bacteremia included male sex, elevated heart rate, low CD4 count, absence of HAART, chief complaint of fever, low serum sodium, and low serum hemoglobin (Table 2). The predictive ability (as measured by AUC) of the final model comprising these predictors was 0.85 (95% CI 0.80–0.89) and remained unchanged with cross-validation [mean AUC = 0.82 (range: 0.61–0.91)].

**Figure 3:**
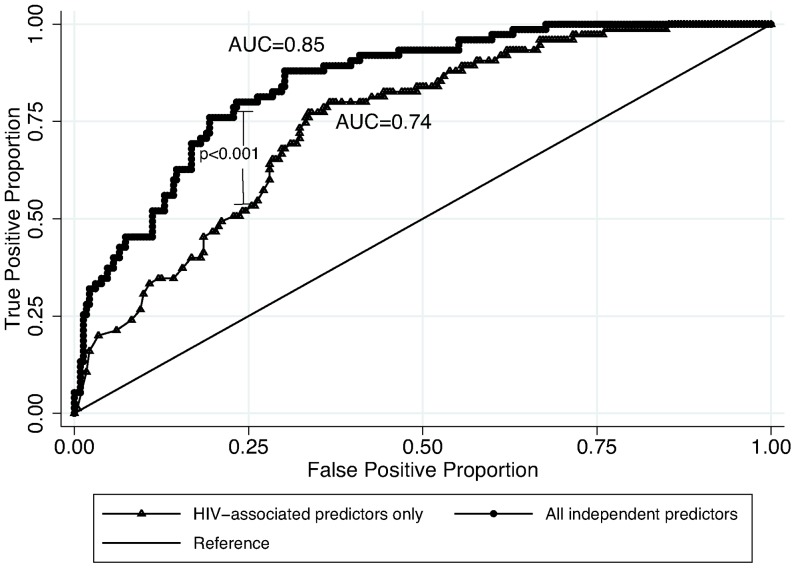
Receiver operating characteristic curves of final logistic regression model containing all independent predictors (sex, CD4 count, HAART status, fever, heart rate, hemoglobin, and sodium) compared to logistic regression model with HIV-associated predictors only (CD4 count and HAART status only) [CD4 = CD4+ T-cell count; HAART = highly active anti-retroviral therapy; AUC = area under the curve].

When compared to a model containing only HIV-associated predictors (i.e., low CD4 count and absence of HAART), the final model substantially improved the predictability of MTB bacteremia with the addition of non-HIV-associated predictors (AUC_HIV model_ = 0.74 vs AUC_final model_ = 0.85, p<0.001) (Figure 3). Also, the model comprised of factors not dependent on laboratory measurements (i.e., male sex, tachycardia, absence of HAART and chief complaint of fever) performed similarly to the HIV-associated predictor model (AUC_HIV model_ = 0.74 vs AUC_without laboratory measurements model_ = 0.70, p = 0.23).

**Figure 4:**
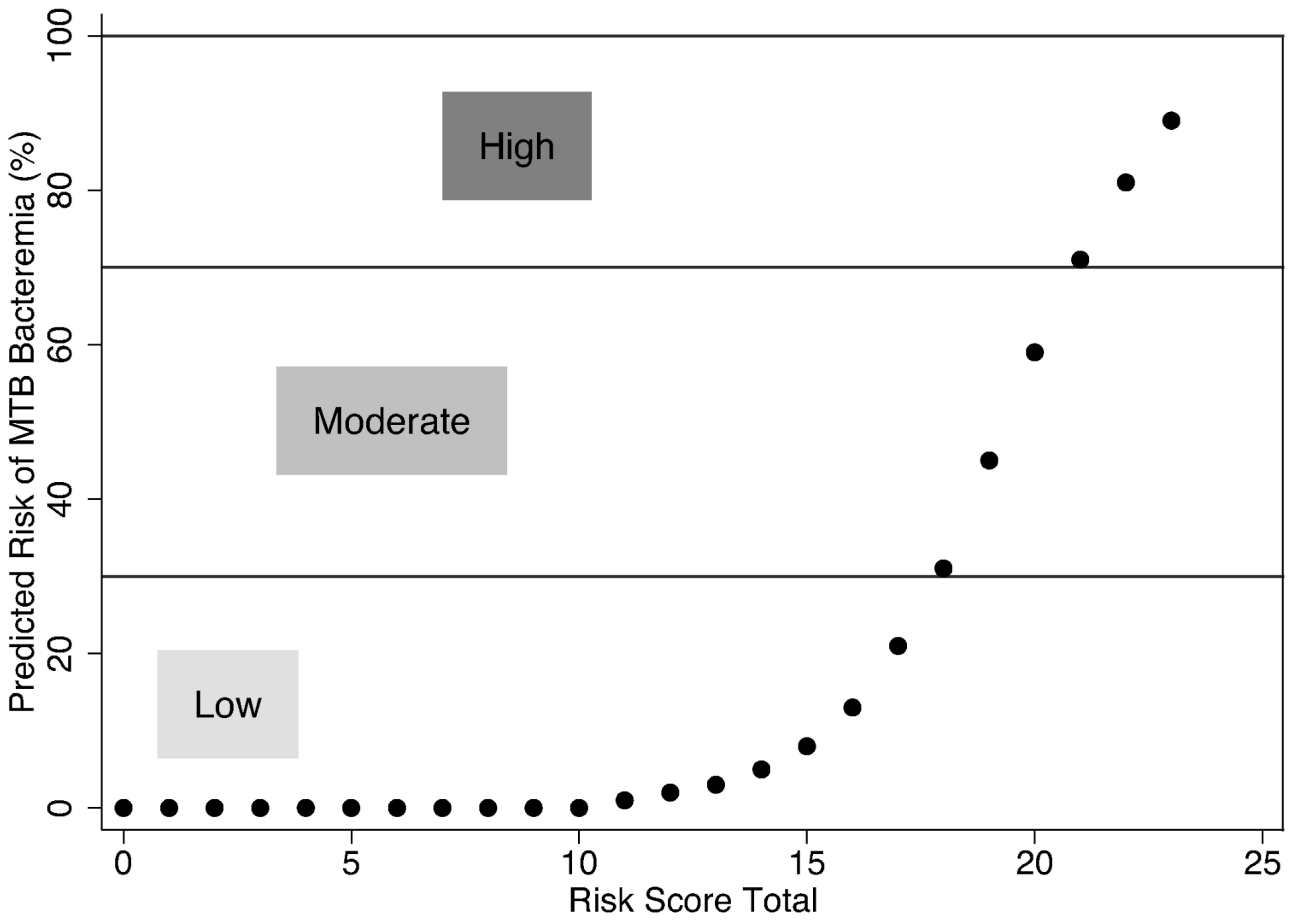
Risk of MTB bacteremia corresponding to MTB bacteremia risk score totals.

A risk score for stratification of the likelihood of having MTB bacteremia was based on points assigned to categories for each predictor comprising the final model (Table 4; Figure 4). Out of the total 25 potential risk score points, non-HIV related factors (tachycardia, chief complaint of fever, anemia and hyponatremia) contributed a similar amount of weight compared to HIV-related factors (CD4 count and HAART use) (Table S2). Sixteen of 284 patients had a MTB bacteremia risk score greater than 21 points which corresponded to a probability of having a diagnosis of MTB bacteremia greater than 70%.

**Table 4:** Patient characteristics and the number of risk score points assigned by the multivariable logistic regression to predict MTB bacteremia.

Category strata	Point Value
***Sex***	
Female	0
Male	2
***HAART***	
Currently taking HAART	0
Not on HAART	4
***CD4 count*** (lymphocytes/mm^3^)	
+200	0
51–199	5
≤50	7
***Primary complaint at Admission***	
Other than fever	0
Fever	1
***Heart Rate*** (beats/min)	
<110	0
110–139	1
≥140	2
***Hemoglobin*** (g/dL)	
>10.5	0
9.5–10.5	3
8.0–9.4	3
6.5–7.9	4
<6.5	5
***Sodium*** (mEq/L)	
>135	0
130–135	1
123–129	2
116–122	3
<116	4

[abbreviations: MTB = *Mycobacterium tuberculosis*; HAART = highly active anti-retroviral therapy; CD4 = CD4+ T-cell count].

## Discussion

In this prospective study of adult patients with severe sepsis admitted to 2 Ugandan hospitals, we show that MTB was isolated from blood in nearly 1 in 4 HIV-infected patients and that these patients had significantly lower 30-day survival than HIV-infected patients without MTB bacteremia. The small proportion of patients who underwent standard MTB diagnostic testing or received empiric anti-tuberculosis therapy suggests that health practitioners in this setting have a low suspicion for diagnosing MTB infection in patients with a primary clinical presentation of severe sepsis.

Previous studies have suggested that standard testing for pulmonary MTB infection may be sufficient for diagnosis of MTB bacteremia. In a study of 344 hospitalized Malawian patients with fever, hypotension or history of recent fever, 103 patients were identified as having any MTB infection, of whom 57 had confirmed MTB bacteremia [8]. Forty-four (77%) patients with MTB bacteremia were identified as having MTB infection using abnormal chest radiograph or sputum AFB smear as a diagnostic test–positive sputum AFB smears had a sensitivity of 32% and abnormal chest radiographs had a sensitivity of 77% for diagnosing MTB bacteremia. Among our cohort patients, a smaller proportion received either an AFB smear (25%) or chest radiograph (53%) suggesting pulmonary MTB was not commonly suspected. Among those who did receive AFB smear and/or chest radiograph, we report a higher sensitivity of AFB smear (77%) and chest radiograph (98%) for diagnosing MTB bacteremia. We also found, however, that the specificity of both sputum AFB (63%) and abnormal chest radiograph (6%) for correctly identifying patients with MTB bacteremia was poor and lower than reported in the Malawi study (82% and 16%, respectively).

The poorer specificity for identifying MTB bacteremia found in the subset of our patient population that received standard MTB diagnostic tests may be a function of the criteria leading clinicians to suspect pulmonary MTB. Accordingly, these patients likely had traditional pulmonary MTB symptoms associated with a positive chest radiograph reflecting pulmonary TB that had not disseminated to the bloodstream. Had all patients included in our study received these diagnostic tests and had those additional test results been negative, the specificity of AFB smear would have increased to a level more comparable to the Malawi study (92.6%), however specificity of chest radiograph would have remained at a sub-optimal level (52.2%). Further, the low specificity in our study and associated high proportion of AFB smears falsely positive for MTB bacteremia could reflect poor quality control for AFB smears if non-tuberculosis mycobacterial infection were erroneously identified as MTB, particularly since 19% of patients with positive AFB sputum smears had non-tuberculous mycobacteria isolated in blood culture. Sputum culture, rather than AFB smear, might correctly identify MTB bacteremia but sputum culture was not systematically obtained in our study patients and would not offer any additional benefit in terms of early diagnosis of MTB bacteremia compared to blood culture.

Patients with MTB bacteremia in our cohort had a high mortality with most deaths occurring within 18 days of admission. Since the majority of results from mycobacterial cultures were not available until after this period, it is clear that the gold standard diagnostic test for accurately identifying HIV-infected patients with MTB bacteremia is inadequate. Recent developments in point-of-care diagnostic assays (e.g., GenXpert, urinary lipoarabinomannan) for MTB infection are promising for diagnosis of MTB bacteremia in resource-constrained settings but validated procedures for blood testing are not presently available [23], [24]. Moreover, data from two studies in Tanzania for the GenXpert assay suggest that the yield in colony forming units (CFU) from blood cultures is considerably lower than the limit of detection threshold (131 CFU/ml) required for diagnosis [11], [25]. Despite the limitations of GenXpert for detecting MTB in blood, GenXpert may still be able to increase detection of MTB cases if sputum samples for GenXpert analysis are collected in all patients with severe sepsis regardless of whether or not respiratory symptoms are present. Ultimately, further diagnostic studies need to evaluate how many patients with undiagnosed pulmonary or extra-pulmonary MTB can be correctly diagnosed with just chest radiograph and AFB smear and determine the role of more expensive tests in improving diagnostic accuracy for early identification of MTB bacteremia.

In the meantime, we present a risk stratification tool adapted from clinical predictors to help clinicians determine a severely ill hospitalized patient’s level of risk for having MTB bacteremia. Using this risk score, we demonstrate that the addition of predictors not directly related to HIV infection (sex, heart rate, fever, sodium and hemoglobin) add substantial predictive accuracy over and above what is contributed by HIV-associated predictors (CD4 count and taking HAART) for identifying HIV-infected patients with MTB bacteremia. Notably, these factors include laboratory-dependent variables (i.e., sodium, hemoglobin and CD4 count) which may limit the generalizability of the risk score model only to settings where the laboratory capacity is sufficient to support measurement of these tests. Nevertheless, current development of point-of-care assays to determine CD4 cell counts and electrolyte levels may improve the feasibility of being able to reliably and regularly measure these factors in resource-limited settings.

Based on our findings, HIV-infected patients hospitalized with a high MTB bacteremia risk score comprising HIV-related and non HIV-related factors have greater than a 70% risk for having a diagnosis of MTB bacteremia. While a larger sample size to validate the MTB bacteremia risk score is necessary before implementation in similar settings, these data suggest that utilization of the factors comprising the MTB bacteremia risk score can help to determine whether more costly diagnostic testing is warranted or whether potentially life-saving treatment should be initiated. Moreover, the MTB bacteremia risk score should be externally validated in other African as well as non-African settings to evaluate the incremental benefit of the MTB bacteremia risk score over standard MTB diagnostic testing. Furthermore, evaluation of novel biomarkers which integrate mechanistic understanding of sepsis related to pathogens like MTB would improve understanding of this condition.

A limitation to this study was the lack of data available in all patients regarding standard diagnostic testing for MTB infection (i.e., chest radiograph and AFB sputum smear). Nonetheless, we provide a realistic estimation of how frequently clinicians might suspect a diagnosis of MTB infection in a patient with a syndrome of severe sepsis in this setting. Moreover, we demonstrate from a multivariable analysis evaluating predictors for initiating empiric anti-tuberculosis therapy that clinicians base their suspicion for MTB infection on whether or not a patient has a cough. While cough is a classic symptom in a case definition for pulmonary MTB infection, we did not find it to be an independent predictor for MTB bacteremia in our cohort. We were also limited by missing predictor data in the development of the MTB bacteremia prediction model and associated risk scores. Given the equal distribution of missing data between patients with and without MTB bacteremia, however, we assume that the occurrence of missing data was unsystematic.

Importantly, utilizing tests with low specificity for MTB bacteremia, like AFB smear and chest radiograph, may result in initiation of anti-tuberculosis therapy. While our data suggest a trend towards improved survival in patients with MTB bacteremia who received empiric anti-tuberculosis therapy, initiation of this therapy can result in morbidity and mortality from drug toxicity which can occur from anti-tuberculosis therapy alone or when interacting with HAART [26]. Although some researchers have suggested an empiric trial of anti-tuberculosis therapy for all febrile hospitalized patients with HIV infection and severe immune suppression in settings like Uganda [27]–[29], the benefit needs to be weighed against potential risks for toxicity and the unclear efficacy of anti-tuberculosis therapy in patients who present emergently with severe sepsis caused by MTB bacteremia. Therefore, further studies should be conducted to determine the safety and efficacy of empiric 4-drug anti-tuberculosis treatment in this context. In the meantime, efforts should be made to increase clinician suspicion for MTB bacteremia among severely ill HIV-infected patients in sub-Saharan Africa and improve the accuracy of early diagnosis using a combination of the MTB bacteremia risk score and other available tests (such as chest radiography, AFB sputum smear and relevant novel diagnostics) to optimize performance characteristics for diagnosis of MTB bacteremia.

## Supporting Information

Table S1 **MTB bacteremia risk score calculations.**
(DOCX)

Table S2 **Estimated probability of MTB bacteremia among HIV-infected patients presenting with severe sepsis and corresponding MTB bacteremia risk score category.**
(DOCX)

Methods S1 **Description of methodology used to derive the MTB bacteremia risk score.**
(DOCX)
