# Correction: *Mycobacterium tuberculosis* Bacteremia in a Cohort of HIV-Infected Patients Hospitalized with Severe Sepsis in Uganda–High Frequency, Low Clinical Suspicion and Derivation of a Clinical Prediction Score

**DOI:** 10.1371/annotation/0a53f994-bfe2-45db-9dbb-97fdfea023c5

**Published:** 2013-08-08

**Authors:** Shevin T. Jacob, Patricia B. Pavlinac, Lydia Nakiyingi, Patrick Banura, Jared M. Baeten, Karen Morgan, Amalia Magaret, Yuka Manabe, Steven J. Reynolds, W. Conrad Liles, Anna Wald, Moses L. Joloba, Harriet Mayanja-Kizza, W. Michael Scheld

In the title, the word "Suspicion" was omitted. The full, correct title can be found below:

*Mycobacterium tuberculosis* Bacteremia in a Cohort of HIV-Infected Patients Hospitalized with Severe Sepsis in Uganda–High Frequency, Low Clinical Suspicion and Derivation of a Clinical Prediction Score

The full, correct citation can be found below:

Jacob ST, Pavlinac PB, Nakiyingi L, Banura P, Baeten JM, et al. (2013) *Mycobacterium tuberculosis* Bacteremia in a Cohort of HIV-Infected Patients Hospitalized with Severe Sepsis in Uganda–High Frequency, Low Clinical Suspicion and Derivation of a Clinical Prediction Score. PLoS ONE 8(8): e70305. doi:10.1371/journal.pone.0070305

