# Supplementary material for: Mycobacterium tuberculosis Bacteremia in a Cohort of HIV-Infected Patients Hospitalized with Severe Sepsis in Uganda–High Frequency, Low Clinical Sand Derivation of a Clinical Prediction Score
Source: PLoS One. 2013 Aug 5;8(8):e70305. doi: 10.1371/journal.pone.0070305 (PMC3734073; doi:10.1371/journal.pone.0070305)
Supplement: Table S1 — MTB bacteremia risk score calculations. (DOCX) [file pone.0070305.s001.docx]

**Table S1. MTB bacteremia risk score calculations**

| **Predictor** | **Reference Value (W*_ij_*)** | **Referent Group**  **(W*_iREF_*)** | **β*_i_*** | **(W*_ij_* – W*_iREF_*)** | **β_i_**  **(*W_ij_* – *W_iREF_*)** | **B** | **Points**  **[β_i_**  **(*W_ij_ -W_iREF_*)**  **/B]** |
| --- | --- | --- | --- | --- | --- | --- | --- |
| **Sex** |  |  | 1.0578 |  |  | 0.5660 |  |
| Female | 0 | Ref |  | 0 |  |  | 0 |
| Male | 1 |  |  | 1 | 1.0578 |  | 2 |
| **HAART** |  |  | 2.1581 |  |  |  |  |
| On HAART | 0 | Ref |  | 0 | 0 |  | 0 |
| No HAART | 1 |  |  | 1 | 2.1581 |  | 4 |
| **CD4 count *** |  |  | -0.0113 |  |  |  |  |
| ≤50 | 24.5 |  |  | -354.5 | 4.0129 |  | 7 |
| 51-199 | 125 |  |  | -254 | 2.8752 |  | 5 |
| 200+ | 379 | Ref |  | 0 | 0 |  | 0 |
| **Heart Rate** |  |  | 0.0168 |  |  |  |  |
| <110 | 90 | Ref |  | 0 | 0 |  | 0 |
| 110-139 | 124.5 |  |  | 34.5 | 0.5802 |  | 1 |
| >=140** | 160 |  |  | 70 | 1.1773 |  | 2 |
| **Sodium** |  |  | -0.0893 |  |  |  |  |
| >135 | 140.5 | Ref |  | 0 | 0 |  | 0 |
| 130-135 | 132.5 |  |  | -8 | 0.714 |  | 1 |
| 123-129 | 126 |  |  | -14.5 | 1.2941 |  | 2 |
| 116-122 | 119 |  |  | -21.5 | 1.9189 |  | 3 |
| <116 | 112 |  |  | -28.5 | 2.5436 |  | 4 |
| **Hemoglobin** |  |  | -0.2767 |  |  |  |  |
| >10.5 | 15.5 | Ref |  | 0 | 0 |  | 0 |
| 9.5-10.5 | 10 |  |  | -5.5 | 1.5219 |  | 3 |
| 8.0-9.4 | 8.7 |  |  | -6.8 | 1.8817 |  | 3 |
| 6.5-7.9 | 7.2 |  |  | -8.3 | 2.2968 |  | 4 |
| <6.5 | 5.4 |  |  | -10.1 | 2.7948 |  | 5 |
| **Fever** |  |  | 0.6897 |  |  |  |  |
| Not primary complaint | 0 | Ref |  | 0 |  |  | 0 |
| Primary complaint | 1 |  |  | 1 | 0.6897 |  | 1 |
| **Intercept** |  |  | 8.6357 |  |  |  |  |
