# Supplementary material for: Mycobacterium tuberculosis Bacteremia in a Cohort of HIV-Infected Patients Hospitalized with Severe Sepsis in Uganda–High Frequency, Low Clinical Sand Derivation of a Clinical Prediction Score
Source: PLoS One. 2013 Aug 5;8(8):e70305. doi: 10.1371/journal.pone.0070305 (PMC3734073; doi:10.1371/journal.pone.0070305)
Supplement: Table S2 — Estimated probability of MTB bacteremia among HIV-infected patients presenting with severe sepsis and corresponding MTB bacteremia risk score category. (DOCX) [file pone.0070305.s002.docx]

**Table S2. Estimated probability of MTB bacteremia among HIV-infected patients presenting with severe sepsis and corresponding MTB bacteremia risk score category**

| **Risk score** | **Estimated probability of MTB bacteremia** | **Risk score category** | **Number of subjects** |
| --- | --- | --- | --- |
| 0 | <0.01 | 1 (low) | 1 |
| 1 | <0.01 | 1 (low) | 2 |
| 2 | <0.01 | 1 (low) | 1 |
| 3 | <0.01 | 1 (low) | 0 |
| 4 | <0.01 | 1 (low) | 2 |
| 5 | <0.01 | 1 (low) | 2 |
| 6 | <0.01 | 1 (low) | 3 |
| 7 | <0.01 | 1 (low) | 3 |
| 8 | <0.01 | 1 (low) | 12 |
| 9 | <0.01 | 1 (low) | 9 |
| 10 | <0.01 | 1 (low) | 10 |
| 11 | 0.01 | 1 (low) | 13 |
| 12 | 0.02 | 1 (low) | 22 |
| 13 | 0.03 | 2 (moderate) | 17 |
| 14 | 0.05 | 2 (moderate) | 19 |
| 15 | 0.08 | 2 (moderate) | 22 |
| 16 | 0.13 | 2 (moderate) | 27 |
| 17 | 0.21 | 2 (moderate) | 30 |
| 18 | 0.31 | 2 (moderate) | 33 |
| 19 | 0.45 | 2 (moderate) | 24 |
| 20 | 0.59 | 2 (moderate) | 16 |
| 21 | 0.71 | 3 (high) | 9 |
| 22 | 0.81 | 3 (high) | 6 |
| 23+ | ≥0.89 | 3 (high) | 1 |
