# Supplementary material for: Mycobacterium tuberculosis Bacteremia in a Cohort of HIV-Infected Patients Hospitalized with Severe Sepsis in Uganda–High Frequency, Low Clinical Sand Derivation of a Clinical Prediction Score
Source: PLoS One. 2013 Aug 5;8(8):e70305. doi: 10.1371/journal.pone.0070305 (PMC3734073; doi:10.1371/journal.pone.0070305)
Supplement: Methods S1 — Description of methodology used to derive the MTB bacteremia risk score. (DOCX) [file pone.0070305.s003.docx]

**METHODS S1**

A points-based system was developed to estimate the probability of MTB bacteremia using previously described methods for deriving risk scores (Table S1) [1]. The following logistic regression model obtained from backwards stepwise modeling was used to determine which variables were to be used in the risk score and the associated regression coefficients (β*_i_*):

logit(probability of MTB bacteremia|x_male,_ x_noHAART,_ x_heart rate,_ x_sodium,_ x_hemoglobin,_ x_fever_)=8.64 + 1.06male + 2.16noHAART - 0.011CD4count + 0.017Heart Rate -0.089sodium -0.28hemoglobin + 0.69fever

Quantitative variables from the model were categorized according to clinically relevant cut-offs and a reference value (W_ij_) operationalized as the midpoint of each category range [2]. In cases when the upper or lower limit of the category range was not specified, the 1^st^ and 99^th^ percentile values were used as the lower and upper limit for midpoint calculations. For nominal variables, 0 and 1 were used as the reference values. A base risk profile [referent group (W*_iREF_*)] was set to correspond to the lowest risk group for each variable. We then determined the distance between each reference value and the corresponding referent group in terms of regression units by multiplying the difference by the β*_i_* coefficient [β*_i_* (W*_ij_* – W*_iREF_*)]. To calculate the points corresponding with each of the risk factor categories, this value was divided by a constant (B). We used the constant value (B) of 0.566 which corresponded to the estimated increase in risk associated with a 50 unit decrease in CD4 count from the model. Although the highest point total possible was 25, the highest point value possible from patients in our dataset was 23. Finally, an estimated probability (risk) was calculated for each point total (Table S2).

1. Sullivan LM, Massaro JM, D’Agostino RB (2004) Presentation of multivariate data for clinical use: The Framingham Study risk score functions. Statist Med 23: 1631-1660.
2. National Institute of Allergy and Infectious Diseases. DMID Clinical Research Policies and Standard Procedure Documents. Available: [www.niaid.nih.gov/LabsAndResources/resources/DMIDClinRsrch/pages/toxtables.aspx](http://www.niaid.nih.gov/LabsAndResources/resources/DMIDClinRsrch/pages/toxtables.aspx). Accessed 3 February 2012.
